# Supplementary material for: Asymmetric Synthesis of Quaternary Hydantoins via a Palladium-Catalyzed Aza-Heck Cyclization
Source: J Am Chem Soc. 2025 Nov 14;147(49):44692–8. doi: 10.1021/jacs.5c16022 (PMC12703750; doi:10.1021/jacs.5c16022)
Supplement: Supplementary file 2 [file ja5c16022_si_002.zip › All NMR FID Files/29/29_CarbonNMR/3/pdata/1/email_EM05-090-1-dry-13C_3_1.pdf]

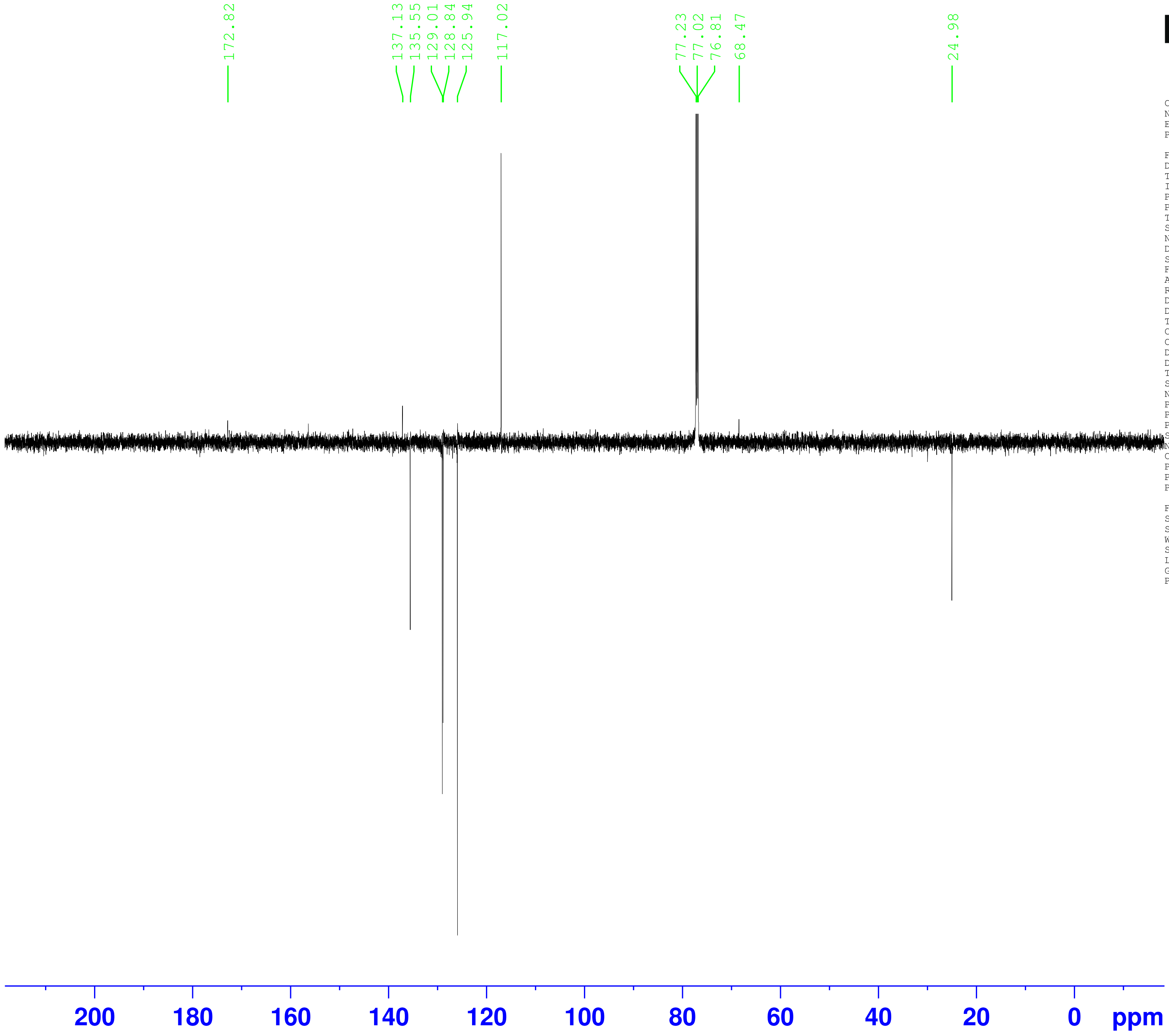

Current Data Parameters
NAME EM05-090-1-dry-13C
EXPNO 3
PROCNO 1

F2 - Acquisition Parameters
Date_ 20240208
Time 23.02 h
INSTRUM Avance Neo 600 at University of Delaware
PROBHD Z185170_0001 (
PULPROG jmod
TD 65536
SOLVENT CDCl3
NS 2048
DS 4
SWH 35714.286 Hz
FIDRES 1.089913 Hz
AQ 0.9175040 sec
RG 101
DW 14.000 usec
DE 19.25 usec
TE 298.0 K
CNST2 145.0000000
CNST11 1.0000000
D1 2.00000000 sec
D20 0.00689655 sec
TD0 1
SFO1 150.9178988 MHz
NUC1 13C
P1 11.78 usec
P2 23.56 usec
PLW1 106.00000000 W
SFO2 600.1324005 MHz
NUC2 1H
CPDPRG[2 waltz65
PCPD2 70.00 usec
PLW2 6.90000010 W
PLW12 0.09034800 W

F2 - Processing parameters
SI 32768
SF 150.9028085 MHz
WDW EM
SSB 0
LB 1.00 Hz
GB 0
PC 1.40
